# Case Report: Clinical Management of a Patient With Metastatic Non-Small Cell Lung Cancer Newly Receiving Immune Checkpoint Inhibition During Symptomatic COVID-19

**DOI:** 10.3389/fimmu.2021.798276

**Published:** 2021-12-20

**Authors:** Sibylle C. Mellinghoff, Kanika Vanshylla, Christine Dahlke, Marylyn M. Addo, Oliver A. Cornely, Florian Klein, Thorsten Persigehl, Jan Rybniker, Henning Gruell, Paul J. Bröckelmann

**Affiliations:** ^1^ Faculty of Medicine and University Hospital of Cologne, Department I of Internal Medicine, Center for Integrated Oncology Aachen Bonn Cologne Düsseldorf (CIO ABCD), University of Cologne, Cologne, Germany; ^2^ Cologne Cluster of Excellence in Cellular Stress Responses in Aging-Associated Disease (CECAD), University of Cologne, Cologne, Germany; ^3^ German Centre for Infection Research (DZIF), Partner Site Bonn-Cologne, Cologne, Germany; ^4^ Department of Clinical Immunology of Infectious Diseases, Bernhard Nocht Institute for Tropical Medicine, Hamburg, Germany; ^5^ Institute of Virology, Faculty of Medicine and University Hospital Cologne, University of Cologne, Cologne, Germany; ^6^ Division of Infectious Diseases, First Department of Medicine, University Medical Center Hamburg-Eppendorf, Hamburg, Germany; ^7^ German Centre for Infection Research (DZIF), Partner Site Hamburg-Lübeck-Borstel-Riems, Hamburg, Germany; ^8^ Clinical Trials Centre Cologne (ZKS Köln), Cologne, Germany; ^9^ Center for Molecular Medicine Cologne (CMMC), University of Cologne, Cologne, Germany; ^10^ Institute for Diagnostic and Interventional Radiology, Faculty of Medicine and University Hospital Cologne, University of Cologne, Cologne, Germany; ^11^ Max-Planck Institute for the Biology of Ageing, Cologne, Germany; ^12^ Mildred-Scheel School of Oncology (MSSO) Aachen Bonn Cologne Düsseldorf, Cologne, Germany

**Keywords:** SARS-CoV-2, COVID-19, NSCLC, anti-PD1, immune checkpoint, immunotherapy, pneumonitis

## Abstract

Effects of initiation of programmed-death-protein 1 (PD1) blockade during active SARS-CoV-2 infection on antiviral immunity, COVID-19 course, and underlying malignancy are unclear. We report on the management of a male in his early 40s presenting with highly symptomatic metastatic lung cancer and active COVID-19 pneumonia. After treatment initiation with pembrolizumab, carboplatin, and pemetrexed, the respiratory situation initially worsened and high-dose corticosteroids were initiated due to suspected pneumonitis. After improvement and SARS-CoV-2 clearance, anti-cancer treatment was resumed without pembrolizumab. Immunological analyses with comparison to otherwise healthy SARS-CoV-2-infected ambulatory patients revealed a strong humoral immune response with higher levels of SARS-CoV-2-reactive IgG and neutralizing serum activity. Additionally, sustained increase of Tfh as well as activated CD4^+^ and CD8^+^ T cells was observed. Sequential CT scans showed regression of tumor lesions and marked improvement of the pulmonary situation, with no signs of pneumonitis after pembrolizumab re-challenge as maintenance. At the latest follow-up, the patient is ambulatory and in ongoing partial remission on pembrolizumab. In conclusion, anti-PD1 initiation during active COVID-19 pneumonia was feasible and cellular and humoral immune responses to SARS-CoV-2 appeared enhanced in our hospitalized patient. However, distinguishing COVID-19-associated changes from anti-PD1-associated immune-related pneumonitis posed a considerable clinical, radiographic, and immunologic challenge.

## Introduction

The coronavirus disease 2019 (COVID-19) pandemic caused by the Severe Acute Respiratory Syndrome Coronavirus-2 (SARS-CoV-2) has a major impact on global health. Cancer patients appear at increased risk for severe COVID-19, especially with cytostatic or B-cell-depleting treatment (1, 2). SARS-CoV-2 infection induces specific antibodies as well as CD4^+^ and CD8^+^ T cells (3). While underlying disease influences antibody kinetics, robust T-cell response is usually also observed with comorbidities (3). Initially, however, a significant T-cell reduction and functional exhaustion of the remaining T cells was observed (4), especially in cancer patients (5). Immune checkpoint inhibition (ICI) reversing T-cell exhaustion could therefore potentially be beneficial for COVID-19 patients. On the other hand, ICI may increase immune hyperactivation during COVID-19, thereby worsening outcomes (6, 7).

The effect of ICI initiation during acute SARS-CoV-2 infection, e.g., by programmed death protein 1 (PD1) blockade, on the course of COVID-19 and development of antiviral immunity remains largely unclear. We report the clinical course, management, and sequential immunological data after first initiation of anti-PD1-based anti-cancer treatment in a patient with newly diagnosed metastatic non-small cell lung cancer (NSCLC) during symptomatic COVID-19. By comparison to otherwise healthy SARS-CoV-2-infected controls (SARS-CoV-2 positive adult individuals with mild disease and without cancer disease), we provide novel insights into the interplay of anti-PD1 treatment with SARS-CoV-2 infection and immunity including potential immune-related adverse events (irAEs).

## Clinical Case Description

A male in his early 40s, who had not received vaccination against SARS-CoV-2, presented to a primary hospital due to progressive severe pain of the lower back and hip accompanied by weight loss. Diagnostics revealed widespread osteolytic lesions with unstable pathologic fractures of the spine and an extensive predominantly right-sided pulmonary tumor. Histopathology revealed NSCLC with relevant PD-Ligand 1 expression (TP-score 10%) but wild type for actionable genes, and staging showed cerebral, hepatic, osseous, peritoneal, pleural, and splenic metastases. During palliative radiotherapy to the spine, a routine nasopharyngeal swab revealed asymptomatic SARS-CoV-2 infection by polymerase chain reaction (PCR; day [D] 0) in the patient who had not received prior COVID-19 vaccination. Due to worsening general condition with Eastern Cooperative Oncology Group (ECOG) score 4, the patient transferred to our department (Timeline: **Table 1**).

**Table 1:** Timeline of the episode of care.

Day 0 (D0)	First nasopharyngeal detection of SARS-CoV-2 by PCR
D0–D7	Progressive cancer-related immobilizing pain and B-symptoms, onset of symptomatic SARS-CoV-2 infection (ECOG 4), and transfer to our department
D8	CT scan reveals progressive metastatic NSCLC and COVID-19 pneumonia, nasopharyngeal swab with high SARS-CoV-2 viral load (qPCR Ct value 18)
D9	First administration of pembrolizumab, pemetrexed, and carboplatin
D10–D15	Peripheral edema, pleural effusion, and ascites judged pemetrexed-related, worsening of respiratory symptoms requiring oxygen supplementation
D16	Initiation of empiric antibiotic treatment with piperacillin/tazobactam due to neutropenic fever, consecutive microbiological diagnostics were negative
D20	CT scan shows changes consistent with worsening COVID-19 pneumonia while treatment-related immune-mediated pneumonitis cannot be ruled out
D20–D25	Empiric treatment with high-dose dexamethasone for suspected pembrolizumab-associated pneumonitis, steady improvement of respiratory symptoms
D27	CT scan shows regressive pulmonary opacities and tumor manifestations
D30	Continuation of anti-cancer treatment with carboplatin and paclitaxel-albumin at standard doses, with regular re-administration after blood count recovery. Concomitant palliative radiotherapy to hip and spine and stereotactic ablation of cerebral metastases.
D47	CT scan shows further regression of tumor lesions and marked improvement of the pulmonary situation, consistent with improved general status
D81	Completion of chemotherapy, initiation of pembrolizumab maintenance treatment
D95	Discharge in an improved overall condition, increasingly mobile with aids and partially capable of self-care (ECOG 2)
6 months	CT scan shows no sign of recurring pneumonitis during ongoing pembrolizumab treatment, and a partial response according to iRECIST is achieved

**Figure 1:**
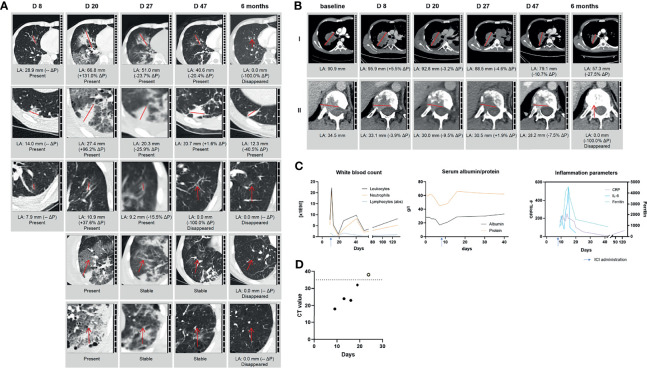
Radiologic examinations and laboratory assessments after initiation of pembrolizumab-based treatment during active COVID-19. **(A)** Exemplary pulmonary infiltrations during SARS-CoV-2 disease course and treatment with pembrolizumab with ground glass infiltration (I+III), consolidation (II), and mixed ground glass and consolidation (IV+V); **(B)** change of the tumor target lesions (I) right upper lobe/hilus and (II) TH12 during SARS-CoV-2 disease course and treatment with pembrolizumab; **(C)** blood counts, inflammatory markers, and serum albumin/protein. **(D)** SARS-CoV-2 viral load reflected by Ct value of SARS-CoV-2 PCR from sequential nasopharyngeal swabs. LA, largest diameter; ΔP, change from previous assessment; DX, day in relation to first SARS-CoV-2 detection by PCR (=D0); CRP, C-reactive protein; IL-6, interleukin 6; LOD, level of detection.

A CT scan on D8 revealed progressive NSCLC and pulmonary infiltrations with mild ground glass opacities as well as mild consolidations attributable to early-stage COVID-19 pneumonia (**Figures 1A, B**). Laboratory examinations showed increased inflammatory markers (**Figure 1C**) and high naso-pharyngeal levels of SARS-CoV-2 RNA (qPCR Cycle threshold [Ct] 18, **Figure 1D**). Due to increase of cancer-related symptoms with immobilizing pain, treatment with pembrolizumab (200 mg Q3W), carboplatin (4 mg/AUC Q3W), and pemetrexed (375 mg/m^2^ BSA) was initiated (D9). Prophylactic dexamethasone 4 mg (1-0-1) was administered peri-chemotherapy and continued at 4 mg/day due to meanwhile symptomatic COVID-19 thereafter.

Within days, the patient developed peripheral edema, pleural effusion, and ascites, judged as primarily pemetrexed-associated. Timely matching serum albumin and serum protein are depicted in **Figure 1C**. Despite adequate fluid management, respiratory symptoms worsened, requiring oxygen supplementation of up to 4 L/min for achieving stable oxygen saturation of >90%. Due to neutropenic fever (**Figure 1C**), empiric antibiotic treatment with piperacillin/tazobactam was initiated on D16; microbiological diagnostics were negative. A D20 CT scan showed progressive opacities consistent with worsening COVID-19 pneumonia but also treatment-related pneumonitis or—less likely—bacterial superinfection (**Figure 1A**) while the tumor manifestations were already regressive (**Figure 1B**). Repeated swabs showed decreasing nasopharyngeal SARS-CoV-2 RNA levels (**Figure 1D**). In light of persisting respiratory symptoms, dexamethasone 20 mg/day was administered (D20–25) for suspected pembrolizumab-associated pneumonitis. Symptoms and inflammatory markers improved subsequently, leading to termination of antibiotic treatment and corticosteroid tapering from D26 onwards, accompanied by regressive tumor and pulmonary opacities on D27 (**Figures 1A, B**).

After blood count recovery and improvement of the general condition, anti-cancer treatment was continued with carboplatin and paclitaxel-albumin (100 mg/m^2^ Q1W) from D30 onwards. Except for prolonged leukopenia, treatment was well tolerated and accompanied by stereotactic radiation of the brain metastasis and palliative radiotherapy for spine and hip. D47 CT showed further regression of tumor lesions and marked improvement of the pulmonary situation with no sign of potential residual immune-related pneumonitis (**Figures 1A, B**). After initiating pembrolizumab maintenance on D81, the patient was discharged on D95 (ECOG 2). The most recent restaging 6 months after treatment start showed a partial response based on iRECIST with a reduced sum of target lesions from 125.4 to 57.3 mm (−54.3%, **Figure 1B**). Notably, there were no signs of active pneumonitis after re-exposure to anti-PD1 treatment. The patient continued ambulatory pembrolizumab maintenance and was increasingly capable of self-care.

## Immune Trajectory

Cellular and humoral immune responses to SARS-CoV-2 were assessed prior to pembrolizumab infusion (D9) and sequentially thereafter (D11, D13, D19, D25, and D39) and compared to otherwise healthy SARS-CoV-2-infected individuals.

**Figure 2:**
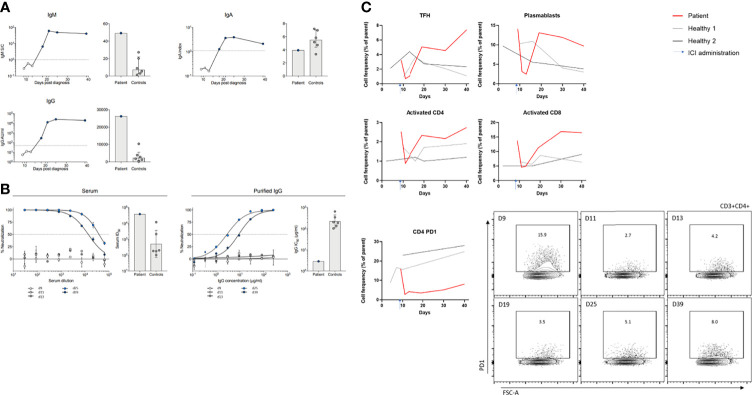
Cellular and humoral immune trajectory during SARS-CoV-2 infection and immune checkpoint inhibition treatment. Cellular and humoral immune responses to SARS-CoV-2 infection were monitored on D9 prior to pembrolizumab infusion, and on D11, D13, D19, D25, and D39 and compared to controls (SARS-CoV-2-positive adult individuals with mild disease and without cancer disease). **(A)** Serum antibodies (IgM, IgA, and IgG) measured by ELISA (IgA, Euroimmun ELISA; IgM and IgG, Abbott Alinity CMIA) targeting SARS-CoV-2 became detectable by D18 post SARS-CoV-2 diagnosis. **(B)** SARS-CoV-2-neutralizing activity was measured, determined by a lentivirus-based pseudovirus neutralization assay as previously described (8) for serum and purified serum IgG from D25 on. Six patients with mild COVID-19 disease served as control for **(A, B)** [right panels; sample acquisition D20 to 30 after PCR confirmation of SARS-CoV-2 infection (8)]. **(C)** Lymphocyte subsets and their expression of activation/differentiation markers (Plasmablasts [(CD20^-^CD38^high^)%CD19^+^], T follicular helper cells [TFH; PD1^+^ICOS^+^], activation status of CD4 and CD8 T cells [HLA-DR^+^CD38^+^], and PD1 expression on CD4^+^ T cells) were determined by flow cytometry. Two subjects with mild COVID-19 disease served as control (sample acquisition for the same period as the patient). Exemplarily, flow cytometry data are shown for CD4^+^ PD1^+^ T cells.

Serum antibodies targeting SARS-CoV-2 became detectable by D18 (**Figure 2A**). High levels of SARS-CoV-2-neutralizing activity were observed in serum as well as for purified serum IgG obtained on D25 and D39 (**Figure 2B**). The appearance of circulating antibodies coincided with decreasing SARS-CoV-2 RNA levels in nasopharyngeal swabs (**Figure 1D**). To further assess the magnitude of the humoral immune response observed on D25 after diagnosis, the findings were contrasted to results obtained within 20–30 days of PCR confirmation of infection from a cohort of overall healthy COVID-19 outpatients with mild disease (*n* = 6), which has been previously described in detail (8). Compared to those, the patient exhibited higher IgM reactivity, 10-fold higher levels of IgG (**Figure 2A**), and 2-logs higher neutralizing serum/IgG activity (**Figure 2B**).

Lymphocyte subsets and their expression of activation/differentiation markers were determined by flow cytometry and compared to two otherwise healthy individuals infected with SARS-CoV-2 (*n* = 2, selected by the following criteria: SARS-CoV-2-positive adult individuals with mild disease and without cancer disease). Similar to antibody kinetics, a high proportion of plasmablasts [(CD20^-^CD38^high^)%CD19^+^] was observed at D18, sustained compared to controls. Of note, the patient had shown a high level of plasmablasts initially that rapidly decreased following ICI treatment. T follicular helper (Tfh) cells (PD1^+^ICOS^+^) showed similar dynamics and initially decreased after ICI initiation, deferredly increasing at D18 (**Figure 2C**). Administration of high-dose corticosteroids entailed stagnation of Tfh levels, but during tapering and thereafter, a further increase beyond the simultaneously measured control patients was found. Furthermore, activated CD4^+^ and CD8^+^ T cells (HLA-DR^+^CD38^+^) showed a similar pattern compared to plasmablasts and Tfh cells. In comparison to Tfh and activated CD4 T cells, CD4^+^ T cells expressing PD1 decreased after ICI initiation and remained low. This pattern was not observed in controls.

## Discussion

We report the clinical and immune trajectory as well as our clinical management approach of a patient with active SARS-CoV-2 infection at the time of pembrolizumab-based treatment initiation for highly symptomatic newly diagnosed metastatic NSCLC. While the effects of SARS-CoV-2 infection in patients already receiving ICI are increasingly studied (9, 10), the effects of a first infusion of an anti-PD1 antibody during active symptomatic COVID-19 remain unknown. Furthermore, the complex case of newly diagnosed cancer patients with urgent need for treatment and concurrent SARS-CoV-2 infection is portrayed. In a situation of rapidly increasing COIVD-19 case numbers as of November 2021, this is highly topical and needs further investigation beyond this case report.

Before treatment initiation in our patient, the risk of potential negative effects of ICI-based anticancer treatment on the COVID-19 disease course was weighed, taking into account a potential benefit of such treatment by reversing T-cell exhaustion. While the patient’s clinical situation initially worsened due to hypervolemia deemed pemetrexed-associated, the tumor manifestations were immediately responsive. Posing a considerable challenge, the patient developed worsening respiratory symptoms accompanied by heterogeneous pulmonary changes not clearly attributable to either COVID-19, bacterial superinfection, or immune-related pneumonitis. While the clinical improvement to corticosteroids hints at an irAE, morphologic features and the lack of pulmonary changes after pembrolizumab re-exposure question this etiology. Hypothetically, ICI-associated pneumonitis may have been aggravated during active SARS-CoV-2 infection due to antigen cross-reactivity and a local state of hyperinflammation.

Despite active cancer and exposure to chemotherapy, our patient showed an adequate humoral immune response with high levels of spike-specific antibodies and potent SARS-CoV-2 neutralizing activity. These values were higher than those seen in temporally matched convalescent individuals with a mild COVID-19 disease course that did not require hospitalization (8). Since mildly ill patients were shown to mount lower binding antibody responses compared to severely ill patients (3, 11), the different course of COVID-19 in our hospitalized patient and the control group has to be taken into account when discussing antibody kinetics. With regard to cellular immune response, we report higher levels of plasmablasts and activated as well as Tfh cells in our patient with ICI administration compared to controls, which is in line with a previous report from a SARS-CoV-2-infected individual under ICI treatment (10). Yet, an influence by the emerging pneumonitis to elevated adaptive immunity, especially with regard to CD4 and CD8 T cells, cannot be ruled out. Due to the lack of measurements in a control group consisting of NSCLC patients under ICI-based treatment without active COVID-19, it remains to be investigated in forthcoming studies to which extent the observed immunologic changes are influenced by initiation of anti-PD1 inhibition during active COVID-19. Immune response in SARS-CoV-2-infected immunocompromised patients has been shown to be frequently impaired, entailing severe COVID-19 courses as well as prolonged viral shedding (1). Correlating viral load, TFH, and plasmablast increase may therefore be additionally reflective of increased germinal center reaction due to antigen load (12). Taking into account the beforementioned circumstances and potential limitations, ICI treatment may possibly have contributed to the patient’s robust humoral and cellular immune response to SARS-CoV-2 infection herein reported. A potentially superior immune response by suppression of the co-inhibitory molecule PD1 however requires substantial further investigation in this context. Besides these immunological observations, our case provides helpful details to inform clinicians managing patients with a first diagnosis of metastatic NSCLC requiring treatment initiation during SARS-CoV-2-related complications.

Due to scarce data, international expert panels strongly recommended to delay/discontinue antineoplastic treatment in SARS-CoV-2-positive tested patients whenever possible (1). On the other hand, uncontrolled malignancy was shown to be a risk factor for severe COVID-19. Initiation or administration of anti-cancer therapy may be highly urgent to preserve vital functions, achieve symptom control, and improve chances of durable remission. In light of the ongoing SARS-CoV-2 pandemic, oncologists must increasingly balance these multifaceted aspects to safely provide optimal antineoplastic care. The here reported case shows feasible and ultimately efficacious treatment initiation with chemo-immunotherapy in an NSCLC patient during acute SARS-CoV-2 infection. The complexity of this clinical course underlines the importance of interdisciplinary teams for treatment of such cases including, among others, oncologists, radiologists, and infectious disease specialists.

## Data Availability Statement

The raw data supporting the conclusions of this article will be made available by the authors upon request, without undue reservation.

## Ethics Statement

The collection of human material involved in this study was reviewed and approved by the Institutional Review Board of the University of Cologne (No 13-091). The patients/participants provided their written informed consent to participate in this study.

## Author Contributions

PB and SM conceptualized the study, organized data and sample collection, analyzed cellular immune response, analyzed data, and wrote the first draft of the manuscript. KV and HG performed the assays on the humoral immune response and co-authored the manuscript. CD, MA, OC, FK, and JR supervised correlative studies and/or patient care and edited the manuscript. TP evaluated radiology images and edited the manuscript. All authors contributed to the article and approved the submitted version.

## Funding

SM is supported by a DZIF clinical leave stipend and PB is supported by the Mildred Scheel School of Oncology (MSSO 202 Postgraduate Program, German Cancer Aid, Grant No. 70113307).

## Conflict of Interest

SM reports personal fees from Octapharma; outside the submitted work. OC reports grants and personal fees from Actelion, personal fees from Allecra Therapeutics, personal fees from Al-Jazeera Pharmaceuticals, grants and personal fees from Amplyx, grants and personal fees from Astellas, grants and personal fees from Basilea, personal fees from Biosys, grants and personal fees from Cidara, grants and personal fees from Da Volterra, personal fees from Entasis, grants and personal fees from F2G, grants and personal fees from Gilead, personal fees from Grupo Biotoscana, personal fees from IQVIA, grants from Janssen, personal fees from Matinas, grants from Medicines Company, grants and personal fees from MedPace, grants from Melinta Therapeutics, personal fees from Menarini, grants and personal fees from Merck/MSD, personal fees from Mylan, personal fees from Nabriva, personal fees from Noxxon, personal fees from Octapharma, personal fees from Paratek, grants and personal fees from Pfizer, personal fees from PSI, personal fees from Roche Diagnostics, grants and personal fees from Scynexis, personal fees from Shionogi, personal fees from Biocon, personal fees from CoRe Consulting, personal fees from Molecular Partners, from MSG-ERC, from Seres, other from Wiley (Blackwell); outside the submitted work. PB received research grants from BeiGene, Bristol Myers Squibb, Merck Sharpe & Dohme, and Takeda; and personal fees and non-financial support from Bristol-Myers Squibb, Celgene, and Takeda; outside the submitted work.

The remaining authors declare that the research was conducted in the absence of any commercial or financial relationships that could be construed as a potential conflict of interest.

## Publisher’s Note

All claims expressed in this article are solely those of the authors and do not necessarily represent those of their affiliated organizations, or those of the publisher, the editors and the reviewers. Any product that may be evaluated in this article, or claim that may be made by its manufacturer, is not guaranteed or endorsed by the publisher.

## References

[1] Giesen N Sprute R Rüthrich M Khodamoradi Y Mellinghoff SC Beutel G. Evidence-Based Management of COVID-19 in Cancer Patients: Guideline by the Infectious Diseases Working Party (AGIHO) of the German Society for Haematology and Medical Oncology (DGHO). Eur J Cancer (Oxford Engl 1990) (2020) 140:86–104. doi: 10.1016/j.ejca.2020.09.009 PMC7505554 33068941

[2] Sullivan RJ Johnson DB Rini BI Neilan TG Lovly CM Moslehi JJ. COVID-19 and Immune Checkpoint Inhibitors: Initial Considerations. J ImmunoTher Cancer (2020) 8(1):e000933. doi: 10.1136/jitc-2020-000933 32434790 PMC7246104

[3] Koch T Mellinghoff SC Shamsrizi P Addo MM Dahlke C. Correlates of Vaccine-Induced Protection Against SARS-CoV-2. Vaccines (2021) 9(3):238. doi: 10.3390/vaccines9030238 33801831 PMC8035658

[4] Diao B Wang C Tan Y Chen X Liu Y Ning L. Reduction and Functional Exhaustion of T Cells in Patients With Coronavirus Disease 2019 (COVID-19). Front Immunol (2020) 11:827. doi: 10.3389/fimmu.2020.00827 32425950 PMC7205903

[5] Abdul-Jawad S Baù L Alaguthurai T Del Molino Del Barrio I Laing AG Hayday TS. Acute Immune Signatures and Their Legacies in Severe Acute Respiratory Syndrome Coronavirus-2 Infected Cancer Patients. Cancer Cell (2021) 39(2):257–75.e6. doi: 10.1016/j.ccell.2021.01.001 33476581 PMC7833668

[6] Indini A Rijavec E Ghidini M Bareggi C Cattaneo M Galassi B. Coronavirus Infection and Immune System: An Insight of COVID-19 in Cancer Patients. Crit Rev Oncol Hematol (2020) 153:103059–. doi: 10.1016/j.critrevonc.2020.103059 PMC7347348 32711241

[7] Moore JB June CH. Cytokine Release Syndrome in Severe COVID-19. Science (2020) 368(6490):473–4. doi: 10.1126/science.abb8925 32303591

[8] Vanshylla K Di Cristanziano V Kleipass F Dewald F Schommers P Gieselmann L. Kinetics and Correlates of the Neutralizing Antibody Response to SARS-CoV-2 Infection in Humans. Cell Host Microbe (2021) 29(6):917–29.e4. doi: 10.1016/j.chom.2021.04.015 33984285 PMC8090990

[9] Luo J Rizvi H Egger JV Preeshagul IR Wolchok JD Hellmann MD. Impact of PD-1 Blockade on Severity of COVID-19 in Patients With Lung Cancers. Cancer Discovery (2020) 10(8):1121–8. doi: 10.1158/2159-8290.CD-20-0596 PMC7416461 32398243

[10] Yatim N Boussier J Tetu P Smith N Bruel T Charbit B. Immune Checkpoint Inhibitors Increase T Cell Immunity During SARS-CoV-2 Infection. Sci Adv (2021) 7(34):eabg4081. doi: 10.1126/sciadv.abg4081 34407944 PMC8373136

[11] Wang Y Zhang L Sang L Ye F Ruan S Zhong B. Kinetics of Viral Load and Antibody Response in Relation to COVID-19 Severity. J Clin Invest (2020) 130(10):5235–44. doi: 10.1172/JCI138759 PMC7524490 32634129

[12] Masiá M Telenti G Fernández M García JA Agulló V Padilla S. SARS-CoV-2 Seroconversion and Viral Clearance in Patients Hospitalized With COVID-19: Viral Load Predicts Antibody Response. Open Forum Infect Dis (2021) 8(2):ofab005. doi: 10.1093/ofid/ofab005 33614814 PMC7881755

